# Rhein and rhubarb similarly protect the blood-brain barrier after experimental traumatic brain injury via gp91^phox^ subunit of NADPH oxidase/ROS/ERK/MMP-9 signaling pathway

**DOI:** 10.1038/srep37098

**Published:** 2016-11-30

**Authors:** Yang Wang, Xuegong Fan, Tao Tang, Rong Fan, Chunhu Zhang, Zebing Huang, Weijun Peng, Pingping Gan, Xingui Xiong, Wei Huang, Xi Huang

**Affiliations:** 1 Laboratory of Ethnopharmacology, Institute of Integrated Traditional Chinese and Western Medicine, Xiangya Hospital, Central South University, 410008 Changsha, China; 2 Department of Infectious Diseases, Key Laboratory of Viral Hepatitis of Hunan, Xiangya Hospital, Central South University, 410008 Changsha, China; 3 Department of Integrated Traditional Chinese and Western Medicine, 2nd Xiangya Hospital, Central South University, 410011 Changsha, China; 4 Department of Oncology, Xiangya Hospital, Central South University, 410008 Changsha, China; 5 Institute of TCM-related Depressive Comorbidity, Nanjing University of Chinese medicine, 210046 Nanjing, China

## Abstract

Oxidative stress chiefly contributes to the disruption of the BBB following traumatic brain injury (TBI). The Chinese herbal medicine rhubarb is a promising antioxidant in treating TBI. Here we performed *in vivo* and *in vitro* experiments to determine whether rhubarb and its absorbed bioactive compound protected the BBB after TBI by increasing ZO-1 expression through inhibition of gp91^phox^ subunit of NADPH oxidase/ROS/ERK/MMP-9 pathway. Rats were subjected to the controlled cortical impact (CCI) model, and primary rat cortical astrocytes were exposed to scratch-wound model. The liquid chromatography with tandem mass spectrometry method showed that rhein was the compound absorbed in the brains of CCI rats after rhubarb administration. The wet-dry weights and Evans blue measurements revealed that rhubarb and rhein ameliorated BBB damage and brain edema in CCI rats. Western blots showed that rhubarb and rhein downregulated GFAP *in vitro*. RT-PCR, immunohistochemistry, Western blot and dichlorodihydrofluorescein diacetate analysis indicated that rhubarb prevented activation of gp91^phox^ subunit of NADPH oxidase induced ROS production, subsequently inhibited ERK/MMP-9 pathway *in vivo* and *in vitro*. Interestingly, rhein and rhubarb similarly protected the BBB by inhibiting this signaling cascade. The results provide a novel herbal medicine to protect BBB following TBI via an antioxidative molecular mechanism.

Traumatic brain injury (TBI) is considered a ‘silent epidemic’ because society is largely unaware of the magnitude of this problem^1^. TBI is the leading cause of long-term disability in children and young adults worldwide^2^. Within the United States, the Centers for Disease Control and Prevention (CDC) estimates that at least 1.4 million people sustain a TBI annually^3^. Of those individuals, 52,000 die and approximately 275,000 are hospitalized^4^. In China, the incidence of TBI is increasing, and the proportion of severe TBI is much higher than the incidence of TBI in other countries^5^. The World Health Organization (WHO) has predicted that TBI will be the third leading cause of global mortality and disability by 2020^6^.

Despite the fact that understanding of the molecular and cellular mechanisms of TBI has improved, many neuroprotective strategies have failed to be translated into a single successful clinical trial or treatment^7^,^8^. There is a need to identify novel chemical entities and drugs to treat TBI^9^. Fortunately, the incorporation of herbal therapy into main stream medical systems has been encouraged by the WHO^10^. Herbal medicine plays a significant role in drug discovery and development^11^. Neuroscientists and doctors hope that novel chemical entities derived from herbal medicines could improve TBI treatment and reduce the risk of mortality and disability^12^.

Rhubarb (*Rheum palmatum* L. or *Rheum tanguticum* Maxim, dahuang in China) is a traditional Chinese herbal medicine that is used as a laxative and stomach drug^13^. It is a highly efficient treatment for TBI patients^14^,^15^. However, the phytochemical from rhubarb that exerts the main neuroprotective effects on TBI patients is unclear. Based on our previous research, rhein (4, 5-Dihydroxyanthraquinone-2-carboxylic acid, Fig. 1A) is the only anthraquinone that is absorbed into the cerebrospinal fluid of TBI patients after rhubarb adminstration^16^. Thus, it is reasonable to propose a hypothesis that rhein derived from rhubarb is responsible for most of the observed neuroprotection following TBI. Rhein has extensive pharmacological effects, including anti-inflammatory, antitumor, antifibrosis, hepatoprotective, nephroprotective, antimicrobial and anti-oxidant activities^17^,^18^,^19^. Unfortunately, the molecular mechanism underlying the protective effects on TBI remains unknown.

The blood-brain barrier (BBB) has the greatest influence on the microenvironment of the brain and plays key roles in promoting optimal neuronal functions including maintenance of brain homeostasis, regulation of influx and efflux transport, and protection from harm^20^. During the acute phase of TBI, BBB damage is a basic pathological change^21^. Owing to the increase in the permeability of the damaged endothelium following BBB disruption, the subsequent brain edema may account for up to half of all observed mortality and morbidity^22^. Therefore, maintenance of BBB integrity to alleviate brain edema is a key goal of TBI treatments.

TBI includes complex biochemical cascades that occur in response to primary and secondary injuries. The events described above immediately generate oxidative stress that is implicated in the development of BBB disruption and brain edema^23^. During oxidation, nicotinamide adenine dinucleotide phosphate (NADPH) oxidase-derived reactive oxygen species (ROS) play crucial roles in BBB breakdown and brain edema^23^,^24^. The main NADPH oxidase subunit expressed in brain tissue is gp91^phox ^25. It maintains enzyme function by producing oxygen radicals. Over-activation of gp91^phox^ in the brain significantly contributes to oxidative damage to neurons^26^. After TBI, gp91^phox^ produces excessive ROS in a “respiratory burst” characterized by O_2_ consumption and, superoxide and hydrogen peroxide production that can, in turn induce the production of more reactive species^27^.

Furthermore, the ROS-mediated activation of extracellular regulated kinase (ERK) plays a central role in the acute phase responses induced by TBI^28^. Following TBI, ROS overproduction through gp91^phox^ activates the ERK1/2 signaling pathway^29^. ERK1/2 is a key component in the activation and expression of matrix metalloproteinase-9 (MMP-9)^30^. MMP-9 is highly expressed in traumatized brains following TBI^31^,^32^. The role of MMP-9 in the BBB impairment has been substantiated^33^. It is involved in extracellular matrix degradation and vascular remodeling which cause the BBB disruption^34^. Phosphorylated ERK1/2 enhances MMP-9 activity and eventually results in BBB dysfunction.

The above pathophysiological processes implicate the gp91^phox^ subunit of NADPH oxidase as a potential source of ROS production after TBI^35^. The activation of gp91^phox^ triggers the ERK/MMP-9 pathway, leading to BBB damage. Inhibition of the gp91^phox^ subunit of NADPH oxidase has been shown to be neuroprotective in acute TBI^23^,^36^. Thus it is reasonable to consider the gp91^phox^ subunit of NADPH oxidase as a therapeutic target to ameliorate BBB damage and brain edema following TBI. Despite the extensive efforts and costs, most antioxidant strategies to treat patients with TBI have failed^37^. More and more scientists and doctors worldwide tend to focus on the use of herbal medicines as antioxidants to protect the BBB after TBI^38^.

In the present study, we performed *in vivo* and *in vitro* experiments to determine whether the antioxidative herbal medicine rhein protected the BBB similar to rhubarb after TBI by suppressing MMP-9 expression through inhibition of the gp91^phox^ subunit of NADPH oxidase/ROS/ERK pathway. We sought to investigate whether rhubarb and its key absorbed compound rhein had potential therapeutic implications for the BBB impairment of TBI.

## Results

### Rhein is absorbed into the brains of CCI rats after intragastric administration of rhubarb

**Figure 1:**
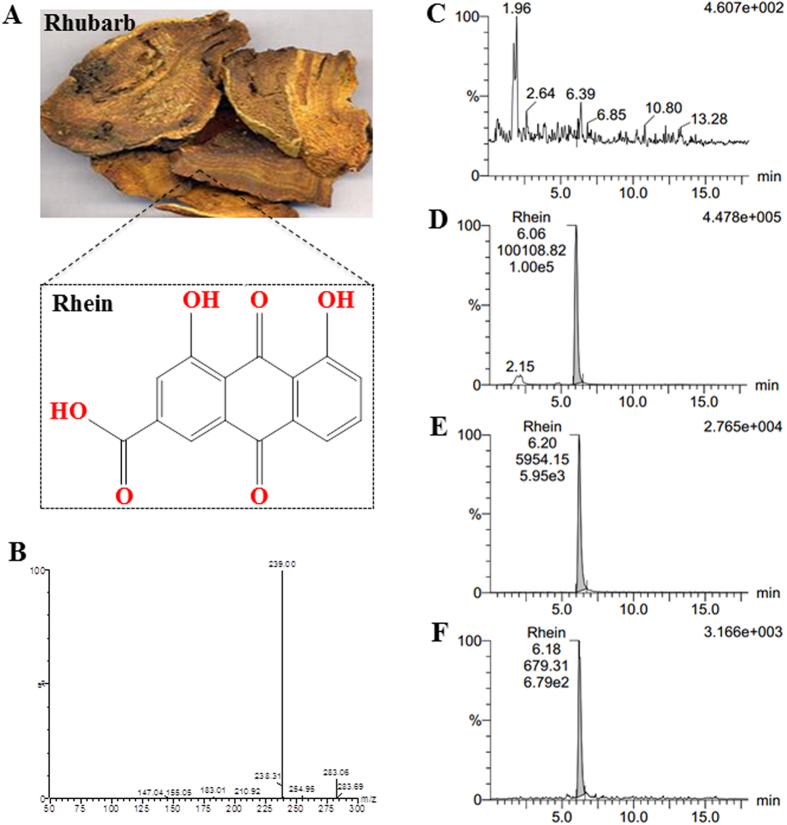
Ultra performance liquid chromatography-electrospray ionization-tandem mass (UPLC-ESI-MS/MS) analysis for determining the rhein content in CCI rats after rhubarb administration. (**A**) The chemical formula of rhein derived from rhubarb. (**B**) LC-MS/MS spectra of rhein, [M-H]^−^ was dominant and used as the precursor ion to obtain the spectra. The mass transitions of rhein were *m/z* 283.06 → 239.0. (**C**) Representative multiple reaction monitoring (MRM) chromatogram of blank brain tissue from a CCI rat. (**D**) Representative MRM chromatogram of rhein originated from its parent herbal medicine rhubarb. (**E**) Representative MRM chromatogram of blank brain tissue from CCI rat spiked with rhein. (**F**) Representative MRM chromatogram of rhein detected in the brain tissue of CCI rat after rhubarb administration.

Rhein is one of main anthraquinones originated from the Chinese herbal medicine rhubarb (Fig. 1A). Using the ultra-performance liquid chromatography-electrospray ionization-tandem mass spectrometry (UPLC-ESI-MS/MS) method^16^, [M-H]^−^ was used as the precursor ion to obtain the ion spectra. The most sensitive mass transitions of rhein were *m/z* 283.06 → 239.0 (Fig. 1B). Representative MRM chromatograms are shown in Fig. 1C–F. The rhein concentration was determined in its parent herb rhubarb which was administered to CCI rats, and the amount of rhein in its parent herb rhubarb was 1.04 ± 0.08 mg/g (Fig. 1D). Furthermore, we found that rhein was absorbed into the brains of CCI rats after oral administration of rhubarb (n = 8/group, Fig. 1E,F).

### Rhubarb and rhein ameliorate BBB damage and brain edema in CCI rats

**Figure 2:**
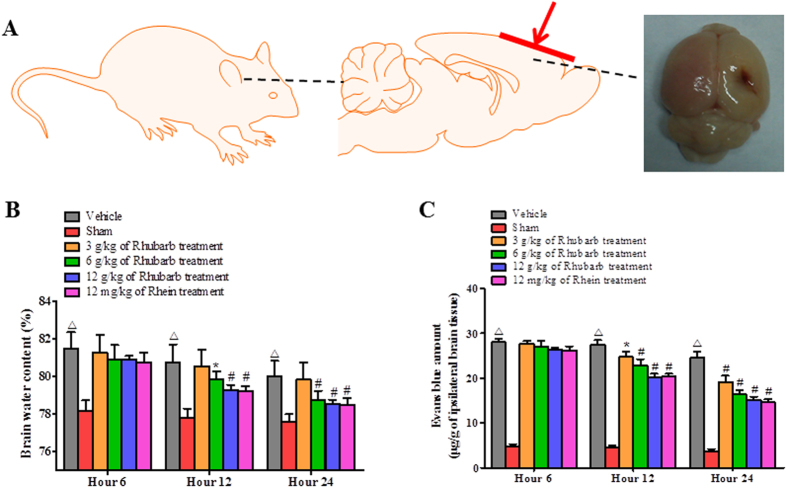
Rhubarb and its absorbed compound rhein attenuate TBI induced BBB permeability and brain edema formation. (**A**) Controlled cortical impact (CCI) models were induced in rats and brain tissues were obtained for the subsequent analyses. (**B**) Effects of rhubarb and rhein on the brain water content in the ipsilateral hemispheres. Compared with the Sham group, the brain water content in the Vehicle group was significantly increased at 6, 12 and 24 h post-CCI. CCI rats that were treated with rhubarb (6 g/kg and 12 g/kg) and 12 mg/kg rhein showed a marked reduction in brain edema at 12 and 24 h post-CCI compared to the Vehicle group. (**C**) The effects of rhubarb and rhein on BBB integrity in the injured hemispheres were assessed using the EB dye extravasation method. Compared with the Sham group, the amount of intracerebral EB dye was significantly increased in the Vehicle group at 6, 12 and 24 h post-CCI. Rhubarb (3 g/kg, 6 g/kg and 12 g/kg) and rhein (12 mg/kg) significantly reduced the quantity of extravasated EB dye at 12 and 24 h post-CCI compared to the Vehicle group. Values are expressed as mean ± SD. n = 6/group, ^Δ^p < 0.01 vs. the Sham group. *p < 0.05 and ^#^p < 0.01 vs. the Vehicle group.

Brains from rats subjected to CCI were used to evaluate BBB permeability and the brain water content (n = 6/group, Fig. 2A). The rhubarb treatment reduced the development of brain edema in the ipsilateral hemisphere in a dose-dependent manner at 12 and 24 h (Fig. 2B). Similarly, rhubarb attenuated TBI-induced Evans Blue (EB) leakage into the brain tissue in a dose-dependent manner at 12 and 24 h compared to the Vehicle group (Fig. 2C). In addition, rhein (12 mg/kg) ameliorated dye extravasation and brain edema, and thus no significant differences were observed compared with the rhubarb (12 g/kg, equivalent to the rhein dosage) treatment (Fig. 2B,C).

### Rhubarb and rhein increase ZO-1 expression by suppressing MMP-9 activation in CCI rats

**Figure 3:**
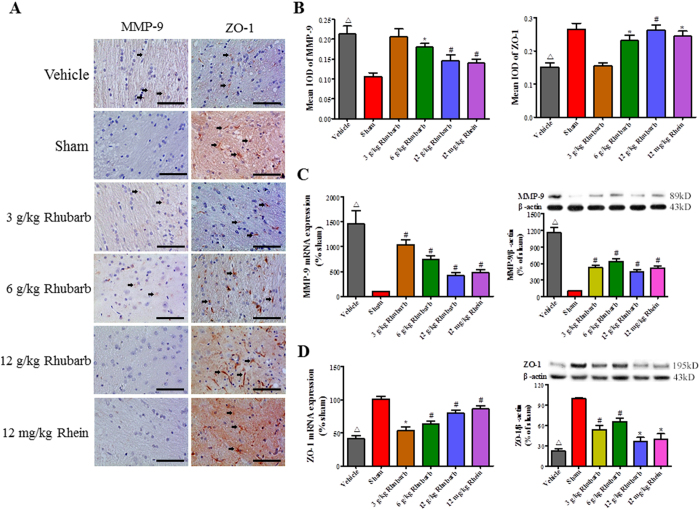
Rhubarb and rhein decrease MMP-9 expression and increased the ZO-1 level in CCI rats at 24 h post-CCI. (**A**) Immunohistochemical examinations of the MMP-9 and ZO-1 positive cells. (**B**) Expression levels in the cortexes of CCI rats were reported as integrated optical density scores. The rhubarb treatments (6 g/kg and 12 g/kg) significantly decreased MMP-9 expression and increased ZO-1 expression compared with the Vehicle group following the cortical contusion. Similarly, 12 mg/kg rhein markedly attenuated MMP-9 expression and increased ZO-1 expression compared with the Vehicle group following the cortical contusion. Scale bar = 200 μm. (**C**) RT-PCR and Western blot analyses of brain MMP-9 and (**D**) ZO-1 expression at 24 h post-CCI. Rhubarb (3 g/kg, 6 g/kg and 12 g/kg) and rhein (12 mg/kg) significantly alleviated the MMP-9 mRNA levels and aggravated the ZO-1 mRNA levels compared with the Vehicle group. In agreement with the RT-PCR results, rhubarb (3 g/kg, 6 g/kg and 12 g/kg) and rhein (12 mg/kg) significantly alleviated the levels of the MMP-9 protein and aggravated the levels of the ZO-1 protein compared with the Vehicle group. The values are expressed as the mean ± SD, n = 6/group, ^Δ^p < 0.01 vs. the Sham group. *p < 0.05 and ^#^p < 0.01 vs. the Vehicle group.

The results of the immunohistochemical examination are shown in Fig. 3A,B (n = 6/group). Enhanced MMP-9 activation and decreased ZO-1 expression were observed in the Vehicle group compared with the Sham group at 24 h. The rhubarb treatment (6 g/kg and 12 g/kg, but not 3 g/kg) decreased MMP-9 expression, accompanied by increased ZO-1 expression. Furthermore, the Western blot (WB) and RT-PCR analyses (n = 6/group) revealed that rhubarb (3 g/kg, 6 g/kg and 12 g/kg) downregulated the expression levels of the MMP-9 mRNA and protein (Fig. 3C), accompanied by elevated the expression levels of the ZO-1 mRNA and protein (Fig. 3D). In addition, rhein (12 mg/kg) exhibited similar performance to 12 g/kg rhubarb by inhibiting both MMP-9 induction and ZO-1 degradation (Fig. 3A–D).

### Rhubarb and rhein block TBI-induced activation of the gp91^phox^ subunit of NADPH oxidase/ROS/ERK pathway in CCI rats

**Figure 4:**
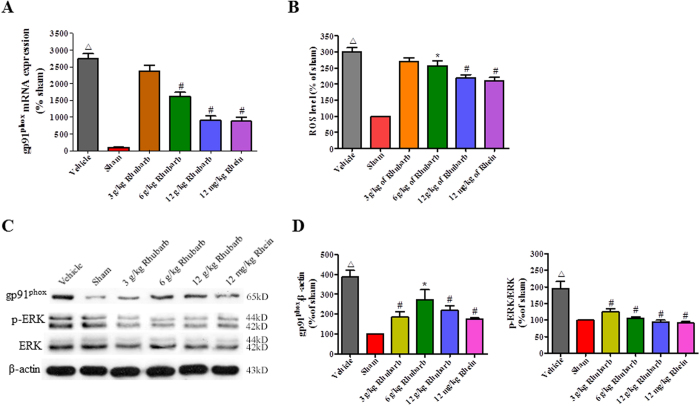
Rhubarb and rhein block TBI-induced activation of the gp91^phox^ subunit of NADPH oxidase/ROS/ERK pathway at 24 h post-CCI. (**A**) Rhubarb (6 g/kg and 12 g/kg) and rhein (12 mg/kg) significantly decreased MMP-9 expression and increased the ZO-1 mRNA levels compared with the Vehicle group at 24 h. (**B**) The analysis of the relative fluorescence intensity showed an increase in ROS production in the ipsilateral cortexes of the Vehicle group compared with the Sham group. The increase was markedly reduced in injured cortexes of CCI rats that were treated with rhubarb (6 g/kg and 12 g/kg) and rhein (12 mg/kg). (**C**) Representative Western blot analysis of the gp91^phox^ expression and the ERK1/2 phosphorylation in the ipsilateral cortexes from CCI rats. (**D**) Quantification of gp91^phox^ expression and ERK1/2 phosphorylation. Rhubarb (3 g/kg, 6 g/kg and 12 g/kg) and rhein (12 mg/kg) suppressed the upregulation of gp91^phox^ expression and ERK1/2 phosphorylation in the ipsilateral cortexes from CCI rats. The values are expressed as the mean ± SD, n = 6/group, ^Δ^p < 0.01 vs. the Sham group. *p < 0.05 and ^#^p < 0.01 vs. the Vehicle group.

In response to TBI, the expression of the gp91^phox^ mRNA and protein was robustly increased in the brains of CCI rats compared with the Sham group (n = 6/group, Fig. 4A,C,D). In addition, ROS production and ERK1/2 phosphorylation were simultaneously upregulated in the brains of CCI rats (Fig. 4B–D). The rhubarb treatment (particularly 6 g/kg and 12 g/kg) blocked the TBI-induced activation of the gp91^phox^/ROS/ERK pathway in the brains of CCI rats (Fig. 4A–D). Additionally, rhein (12 mg/kg) exerted similar gp91^phox^/ROS/ERK inhibition as 12 g/kg rhubarb (Fig. 4A–D).

### Rhubarb and rhein exert a similar pharmacological action as the NADPH oxidase inhibitor apocynin by suppressing the gp91^phox^ subunit of NADPH oxidase/ROS/ERK/MMP-9 signaling pathway in CCI rats

**Figure 5:**
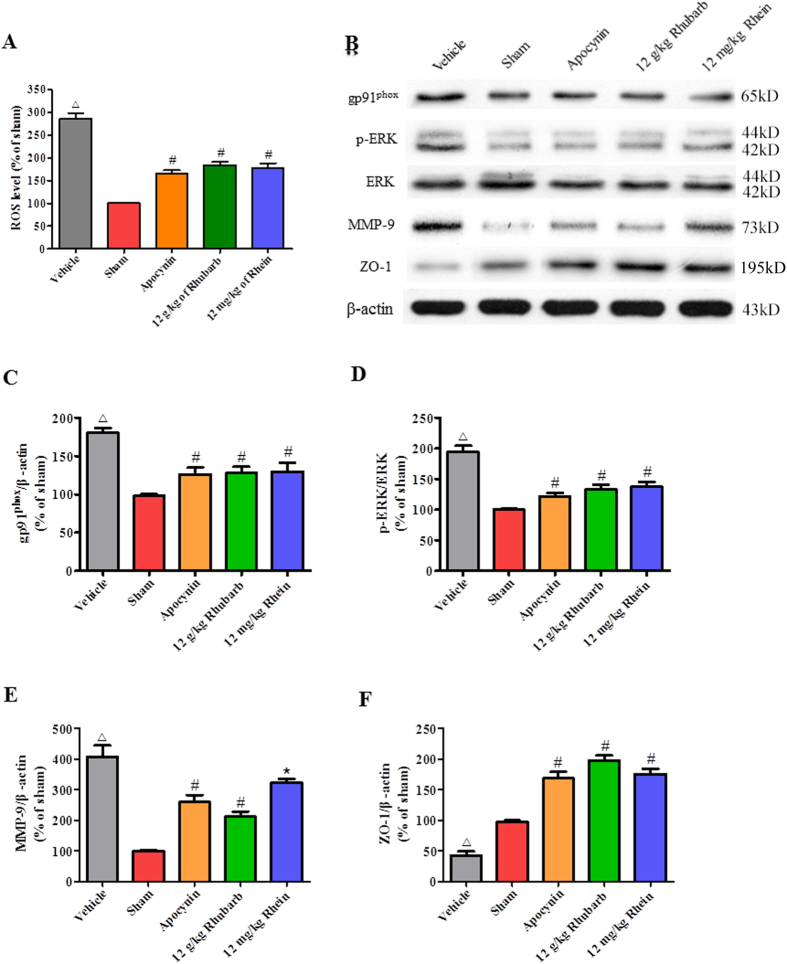
Rhubarb and rhein exert similar pharmacological actions as the NADPH inhibitor apocynin by inhibiting the gp91^phox^ subunit of NADPH oxidase/ROS/ERK/MMP-9 signaling pathway in CCI rats. (**A**) Inhibition of NADPH oxidase with apocynin (50 mg/kg) significantly reduced ROS generation in injured brain cortexes of CCI rats compared with the Vehicle group. Rhubarb and rhein exerted inhibition similar to the NADPH inhibitor apocynin by decreasing ROS production. (**B**) Representative WB analysis of gp91^phox^, p-ERK1/2, ERK1/2, MMP-9 and ZO-1 expression in the ipsilateral cortexes from CCI rats. (**C**) Quantification of the WB showed that inhibition of NADPH oxidase with apocynin (50 mg/kg) significantly downregulated gp91^phox^ and (**D**) MMP-9 expression, (**E**) suppressed ERK1/2 phosphorylation, (**F**) and increased the ZO-1 levels. The values are expressed as the mean ± SD, n = 6/group, ^Δ^p < 0.01 vs. the Sham group. *p < 0.05 and ^#^p < 0.01 vs. the Vehicle group.

After the rats underwent CCI (n = 6/group), gp91^phox^ expression was upregulated in the brains of CCI rats at 24 h (Fig. 5B,C), followed by the activation of ROS/ERK signaling (Fig. 5A,D). The activation of this pathway induced MMP-9 activation (Fig. 5E), leading to ZO-1 degradation (Fig. 5F). Inhibition of NADPH oxidase with apocynin (50 mg/kg) significantly attenuated gp91^phox^ expression and induced the activation of the ROS/ERK/MMP-9 cascade, resulting in ZO-1 upregulation (Fig. 5A–F). Similarly, the inhibitory effects of rhubarb (12 g/kg) and rhein (12 mg/kg) were also detected (Fig. 5A–F).

### The rhubarb and rhein treatments increase ZO-1 expression in scratch-induced rat astrocytes by blocking the gp91^phox^ subunit of NADPH oxidase/ROS/ERK/MMP-9 pathway

**Figure 6:**
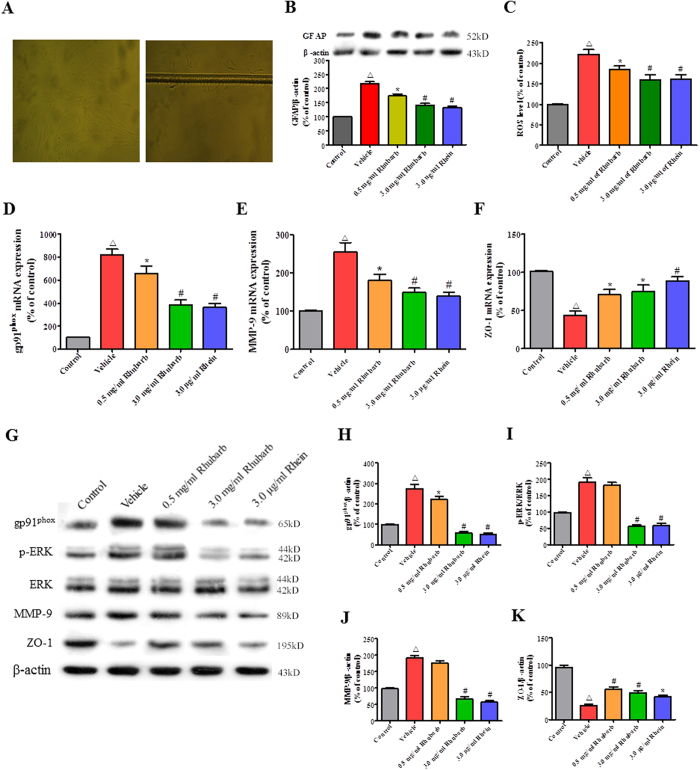
The rhubarb and rhein treatments increase ZO-1 expression in scratch-wounded rat astrocytes by inhibiting the gp91^phox^ subunit of NADPH oxidase/ROS/ERK/MMP-9 pathway. (**A**) Microscopic images of normal and scratch-wounded rat astrocytes. (**B**) The WB analysis showed that rhubarb and rhein reinforced neuroprotection by significantly downregulating the GFAP protein levels. (**C**) ROS production was increased within 24 h in the Vehicle group compared with the Control group. Rhubarb and rhein markedly reversed this trend in the scratch-wounded rat astrocytes. (**D**) The RT-PCR analysis showed that rhubarb and rhein significantly alleviated gp91^phox^ and (**E**) MMP-9 mRNA levels, and (**F**) aggravated the ZO-1 mRNA levels compared with the Vehicle group. (**G**) Representative WB analysis of gp91^phox^, p-ERK1/2, ERK1/2, MMP-9 and ZO-1 expression in the scratch-wounded rat astrocytes. (**H**) Quantification of the WB indicated that rhubarb and rhein significantly decreased the gp91^phox^, (**I**) phosphorylated ERK1/2 and (**J**) MMP-9 levels, and subsequently increased (**K**) ZO-1 expression in the scratch-wounded rat astrocytes. The values are expressed as the mean ± SD, n = 6/group, ^Δ^p < 0.01 vs. the Control group. *p < 0.05 and ^#^p < 0.01 vs. the Vehicle group.

The rat astrocytes were stimulated with a scratch wound (n = 6/group, Fig. 6A), and the subsequent WB analysis revealed that GFAP expression was significantly reduced 24 h after the cells were treated with rhubarb (0.5 and 3.0 mg/mL) and rhein (3.0 μg/mL) compared with the Vehicle group (Fig. 6B). In agreement with our *in vivo* study, a 3.0 mg/mL rhubarb treatment prevented scratch-induced activation of the gp91^phox^/ROS/ERK cascade, subsequently resulting in downregulation of the MMP-9 expression and upregulation of the ZO-1 level in our *in vitro* study (Fig. 6C–K). Meanwhile, treatment with rhein (3.0 μg/mL) exerted similar pharmacological effects as rhubarb (3.0 mg/mL, equivalent to the rhein dosage) through the pathway described above (Fig. 6A–K).

## Discussion

In the present study, we confirmed that rhubarb substantially attenuated BBB damage and brain edema in CCI rats. Both *in vivo* and *in vitro* experiments revealed that rhubarb provided this neuroprotection by increasing ZO-1 expression through suppressing TBI-induced activation of the gp91^phox^ subunit of NADPH oxidase/ROS/ERK/MMP-9 signaling pathway. Interestingly, rhein provided similar BBB protection to rhubarb (equivalent to the rhein dosage) by inhibiting this cascade. Therefore, rhubarb may represent a potential therapeutic agent to protect the BBB and treat TBI. Furthermore, rhein was the absorbed bioactive anthraquinone compound from rhubarb that prevented the BBB disruption after TBI.

Disruption of BBB is required for the development of brain edema, which accounts for more than half of all deaths following severe TBI^39^. BBB stabilization with neuroprotectants may improve functional outcomes after TBI^40^. Strategies to therapeutically engage the BBB repair processes after TBI could also have many beneficial downstream effects^41^. Thus maintenance of BBB integrity constitutes a potential target for brain protection in TBI. However, the current therapeutic strategy used to protect BBB and treat TBI has failed^42^. Researchers are exploring the role of Chinese herbal medicines as a therapeutic strategy to protect BBB following TBI^43^. In this study, the Chinese herb rhubarb and its absorbed bioactive compound rhein markedly ameliorated BBB damage and the development of brain edema in CCI rats. Furthermore, an *in vitro* experiment showed that rhubarb and rhein reinforced neuroprotection by downregulating a cellular index of injury reflected by GFAP expression^44^, which was related to the severity of brain injury and outcomes^45^.

Oxidative stress is believed to be one of the three major deleterious pathways that occur after TBI^46^. During the acute phase of TBI, oxidative stress is the key factor inducing BBB breakdown or paracellular permeability^47^. TBI causes oxidative stress, and ROS overproduction through activation of gp91^phox^ containing NADPH oxidase contributes to the BBB damage in response to TBI. NADPH oxidase is the first enzyme that was shown to intentionally generate ROS in mammalian cells^27^. The catalytic subunit of NADPH oxidase gp91^phox^ is an integral protein containing both a flavin adenine nucleotide and a heme group. Following TBI, gp91^phox^ is immediately upregulated and, significantly amplifies ROS generation through the oxidation of macromolecules and subsequent modulation of redox signaling pathways^23^,^24^. Subsequently, ROS activates enzymes and signaling cascades that regulate lipids and chromatin by inducing ERK1/2-mediated MMP-9 activation and ZO-1 degradation, eventually resulting in BBB dysfunction. NADPH oxidase inhibition reduces brain edema induced by cold brain injury and controlled cortical impact^48^. Targeting the gp91^phox^ subunit of NADPH oxidase-derived ROS production may provide a novel therapeutic strategy for combating BBB disruption following TBI^36^.

**Figure 7:**
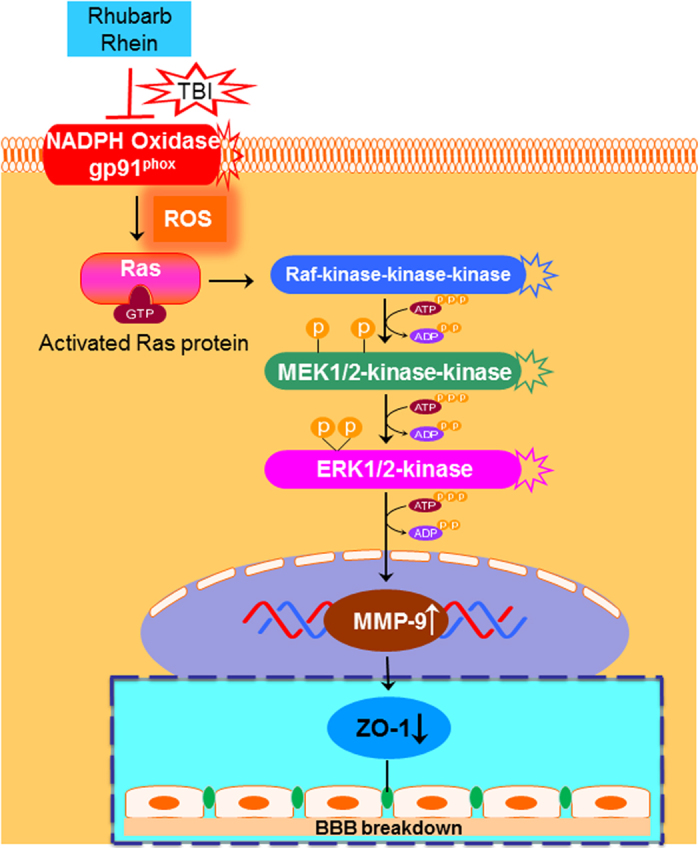
Schematic depiction of the mechanism by which rhubarb and its bioactive component rhein may protect the BBB after TBI via the gp91^phox^ subunit of NADPH oxidase/ROS/ERK/MMP-9 pathway. Trauma is responsible for inducing sudden biochemical changes that occur at the time of impact. During TBI, gp91^phox^ is immediately upregulated. Subsequently, ROS overproduction by NADPH oxidase contributes to BBB damage and brain edema formation through the activation of downstream signaling molecules. After TBI, ROS activates ERK1/2, inducing increased MMP-9 synthesis and ZO-1 degradation. Inhibition of gp91^phox^-triggered ROS production may help prevent the disruption of the BBB and brain edema post-TBI. In the present study, rhubarb is reported to be an effective inhibitor of gp91^phox^-derived ROS production as a treatment for TBI. The underlying mechanisms include ROS-mediated ERK1/2 induction, followed by MMP-9 degradation and increased ZO-1 expression. Furthermore, rhein exerted similar neuroprotective effects as its parent herb rhubarb through the signaling pathway described above.

As shown in Fig. 7, after TBI, the brain is vulnerable to oxidative stress mediated damage that may be successfully treated if therapy is started promptly^49^. Our results revealed that rhubarb and rhein reduced the MMP-9 level and, increased ZO-1 expression *in vivo* and *in vitro*. Furthermore, an analysis of the underlying mechanisms by which the BBB is protected after TBI through MMP-9 inhibition and ZO-1 raise is required, with a particular focus on the signaling pathways^50^. Thus, we evaluated the effects of rhubarb and rhein on the signaling molecules involved in regulating the gp91^phox^ subunit of NADPH oxidase/ROS/ERK cascade. The data from *in vivo* and *in vitro* studies suggested that NADPH oxidase inhibitor apocynin significantly suppressed ROS production, ERK1/2 activation and BBB breakdown after TBI. Similarly, rhubarb and rhein blocked the activation of gp91^phox^, which subsequently inhibited ROS-induced ERK signaling. The results indicated that rhubarb and rhein may serve as a potential therapeutic agent to protect the BBB in the treatment of TBI. However, to confirm above ROS-induced ERK signaling pathway, Ras-Raf -ERK1/2 pathway participate in the progression of herbal action should be further investigated in the future research.

It is notable that there is increasing convergence between traditional Chinese medicine and modern medicine^51^. Our knowledge of the use of herbal medicine has grown empirically over several millennia through experience and folklore, but the products are often indicated for the treatment of a wide variety of seemingly unrelated symptoms, without reference to a mechanism of action or the effects on an underlying disease-causing mechanism^52^. With the development of modern technology, it has become possible to determine the pharmacology and mechanisms of action of many Chinese herbs. Using the UPLC-MS/MS method, we identified rhein as the anthraquinone compound that was absorbed into the brains of CCI rats, which was consistent with our previous clinical research^16^. Furthermore, when the content of rhein was equal to the dosage of rhein in rhubarb, rhein provided similar BBB protection as its parent herb rhubarb following TBI by increasing ZO-1 expression via inhibition of the gp91^phox^ subunit of NADPH oxidase/ROS/ERK/MMP-9 signaling pathway both *in vivo* and *in vitro*. The results may provide evidence that the absorbed bioactive compound from rhubarb exerts the BBB protection and may be used to treat TBI.

The BBB constitutes a component of the neurovascular unit formed by specialized brain endothelial cells surrounded by astrocytes, pericytes and neurons. Reactive astrocytes contribute to increased oxidative stress and the development of brain edema, thus exacerbating secondary brain injury following TBI^53^. Astrocytes determine the brain’s vulnerability to oxidative injury and form a tight functional unit with neurons. An imbalance in the astrocytic energy metabolism, impaired antioxidant capacity and astrocytes death may critically impair neuronal survival^54^. In clinical settings, astrocytes damage induced by TBI adversely affects patient outcomes^55^. Therefore, it is crucial to protect astrocytes from oxidative stress to maintain brain function after TBI^56^. However, the contribution of reactive astrocytes to TBI is a relatively unexplored area of research and provides an additional therapeutic target for TBI treatment^55^. This study used astrocytes as *in vitro* model to investigate BBB protection induced by the rhubarb and rhein treatments following TBI. The *in vitro* results were consistent with the results of our *in vivo* study.

Unlike conventional drugs, the therapeutic effects of traditional Chinese herbal medicines impact multiple molecules and pathways in a biological system. Further research should focus on the mechanisms of multiple targets to examine the neuroprotective effects of rhubarb and its main absorbed bioactive components.

In summary, the findings from our *in vivo* and *in vitro* studies may indicate that rhein provides similar neuroprotection to rhubarb by inhibiting the gp91^phox^ subunit of the NADPH oxidase/ROS/ERK/MMP-9 signaling pathway and subsequently attenuating BBB disruption in response to TBI. The present data demonstrate that rhein is the absorbed bioactive anthraquinone compound of rhubarb that maintains BBB integrity during TBI treatment. This study reports that rhein originated from rhubarb may be an effective compound for development as a potential therapeutic agent to protect the BBB following TBI.

## Methods

### Rhubarb and rhein preparation

Dried rhubarb (voucher specimen No. 20120312, Gansu, China) was obtained from the pharmacy of Xiangya Hospital, Hunan province, China. The plant was authenticated by the herbal medicine botanist Professor Suiyu Hu, Department of Herbal Medicine of Central South University in China. The reference standard for rhein (authorized purities > 98) was purchased from Chengdu Must Biotechnology Company (Chengdu, China).

### Animal preparation

The protocol was approved by the Medical Ethics Committee of Xiangya Hospital of Central South University. The animal experiments were performed according to the guidelines for the care and use of animals established by Central South University. Adult male Sprague-Dawley (SD) rats (age, 8–10 weeks; weight, 200–250 g, Changsha, China) were housed under identical conditions (room temperature at 25 °C, 12-hour light-dark cycle, and 50 ± 10% relative humidity) and had free access to a standard rodent diet and water.

### Controlled cortical impact (CCI) model in rats

The animals were subjected to controlled cortical impact (CCI) model. Briefly, the rats were anesthetized with 3% pentobarbital sodium (50 mg/kg) through an intraperitoneal injection. CCI model was induced with an electronic controlled pneumatic impact device (TBI 0310, precision systems and instrumentation, Fairfax Station, VA) equipped with a hard stop bimba cylinder (Bimba Manufacturing, Monee, IL) and an impactor tip (external diameter of 5.0 mm). This device used an electromagnetic force to produce an impact velocity, and the speed, depth, and dwell time were individually manipulated to produce injuries of different severity. The rats were placed on a stereotaxic frame with a built in heating bed that maintained the body temperature at 37 °C. The rat’s head was mounted in the stereotaxic frame. Under aseptic conditions, a midline longitudinal incision was created in the skull, a 5.0 mm craniotomy was generated over the left parietal cortex (the center of the coordinates of craniotomy relative to bregma: 3.0 mm posterior, 2.5 mm lateral) using a portable drill and trephine, and the bone flap was removed. The rats were then subjected to CCI using a pneumatic cylinder with a 3.0 mm flat-tip impounder, velocity 6.0 m/secs, set depth of 5.0 mm, and dwell time of 100 ms. The Sham injury control rats underwent an identical surgical procedures, but not the cortical impact. The body temperature of the rats was monitored throughout surgery, and a heated cage was used to maintain the body temperature at 37.0 ± 0.5 °C. Approximately 25 min was required for the subjects to fully recover from the operation, and the survival rate after the surgery was greater than 90%.

### Identification of rhein derived from rhubarb in the brains of CCI rats by Liquid Chromatography with tandem mass spectrometry (LC-MS/MS)

The ipsilateral cortex of each rat (n = 8/group) was dissected and stored at −80 °C until homogenization. Each tissue sample, which weighed 0.2 g and surrounded the injury region, was manually homogenized in 9 volumes (1:9, *w/v*) of 0.1 mol/L PBS (pH 7.4) containing 0.01 mol/L Tris-HCl, 0.0001 mol/L EDTA-2Na, 0.01 mol/L cane sugar, and 0.8% normal saline. After the samples were centrifuged at 15000 × g at 4 °C for 15 min, each homogenate was separately evaporated to dryness under nitrogen at 37 °C. Four milliliters of diethyl ether (containing 70 μL of 1 M perchloric acid) was added to each dry extract and vortexed for 60 s. Each dry extract was centrifuged at 15 000 × g at 4 °C for 15 min. The supernatant was transferred to a new tube and dried under nitrogen gas. Any remaining residue was dissolved in 100 μL of pure methanol and then centrifuged at 15 000 × g at 4 °C for 15 min. The upper layer was collected and filtered through a 0.22 μm nylon filter membrane. Finally, 5 μL of the filtrate was injected for the LC-MS/MS analysis.

A Waters Acquity ultra performance liquid chromatography (UPLC) system (Waters Corporation, Milford, MA, USA) coupled to a Waters TQD triple quadruple tandem mass spectrometer was used for the analysis. Chromatographic separation was performed on an Acquity UPLC BEH C18 column (50 × 2.1 mm i.d., 1.7 μm). The mobile phase was composed of methanol and 0.1% formic acid-deionized water with a gradient elution (0 min, 45:55; 15 min, 75:25). The flow rate and operating temperature were maintained at 0.25 mL/min and 35 °C, respectively. The wavelength was 254 nm in the UV spectrum. The autosampler was conditioned at 4 °C.

For operation in MS/MS mode, the Waters Acquity TQD triple quadruple tandem mass spectrometer (Waters Corporation, Manchester, UK) equipped with an ESI interface was connected to the UPLC system. The ESI source was operated in negative mode with the capillary voltage set at 2.5 KV. The desolvation temperature was set at 365 °C. The source temperature was fixed at 110 °C. Nitrogen was used for the desolvation gas flow (650 L/h) and cone gas flow (50 L/h). Argon was used as the collision gas at a flow rate of 0.2 mL/min. Multiple reaction monitoring (MRM) mode was applied for quantification. All data were acquired and processed using Masslynx^TM^ 4.1 software (Waters Corporation).

### Cell culture and scratch wounded model

Primary rat cortical astrocytes were purchased and maintained according to the manufacturer’s protocol (OriCell^TM^, No. SCCAC-0001, Cyagen, USA). The astrocytes were resuscitated at 37 °C, seeded on coverslips in six-well plates and cultured in complete medium (OriCell^TM^ SD Rat Cortical Astrocytes Medium, No. SCCAC-90011, Cyagen, USA). The cells were further cultured in a incubator at constant temperature of 37 °C with 5% CO_2_ and 95% air. The cell culture medium was replaced with fresh medium every two days until the astrocytes reached 90% confluence.

An *in vitro* scratch assay was used for the injury of rat cortical astrocytes as previously described^57^. The scratch-wound model is a mechanical injury model in which a monolayer of confluent astrocytes is ‘wounded’ by scratching with a sterile pipette tip^58^. Parallel scratches were first generated using a sterile pipette tip, and then more scratches were created at a right angle to the previous scratches. After all the floating cells and debris were removed, the astrocytes were kept in an incubator with fresh medium and allowed to recover for 6 h.

### Brain water content assay

Brain edema was assessed using the wet-dry weight method at 6, 12 and 24 h after TBI. Briefly, the rats were sacrificed by decapitation under deep anesthesia. Their brains were quickly removed and separated into left and right hemispheres though the interhemispheric fistula. Tissue samples from the injured hemispheres were placed in glass petri dishes and weighed to obtain the wet weight. The dishes were then baked at 100 °C for 24 h, and reweighed to obtain the dry weight. The percentage of water was calculated using the following formula: [(wet weight-dry weight)/wet weight] × 100.

### BBB permeability evaluation

Because TBI can disrupt the BBB, BBB integrity was examined using the the EB assay. The degree of BBB breakdown was quantitatively evaluated by assessing EB dye leakage. Briefly, an EB dye solution (2% in saline, 2 mL/kg) was injected into the rat’s tail vein for 1 min and allowed to circulate for 2 h prior to sacrifice. The brain was transcardially perfused with 250 mL of saline until a colorless perfusion fluid was obtained. The injured hemisphere was rapidly extracted and weighed. The samples were then incubated in formamide (1 mL/100 mg; Sigma-Aldrich) for 48 h at 60 °C. The absorbance of the supernatant was measured with a spectrophotometer at 620 nm. The quantitative calculation of the dye content in the brain was quantified from a standard curve derived from known amounts of dye and was expressed per gram of tissue.

### Immunohistochemical staining

Immunohistochemistry was performed to detect the BBB impairment during the acute phase after TBI. Briefly, after anesthetization with 3% pentobarbital sodium (100 mg/kg), the brains of rats were removed and stored in 4% paraformaldehyde until processing. Coronal sections were cut on a cryostat at 10 μm thickness, deparaffinized and rehydrated. Sections were permeabilized with 3% H_2_O_2_ for 10 min and blocked with 5% normal donkey serum in PBS for 60 min at room temperature. Immunostaining was performed by incubating the sections with primary antibodies (antibodies are listed in Table 1) against ZO-1, MMP-9 and gp91^phox^ at 4 °C overnight, followed by staining with biotin-labeled secondary antibodies for 120 min and incubation with an avidin-biotin-peroxidase complex (1:100, Sigma, USA) for 1 h at 37 °C. Immunoreactivity was visualized with diaminobenzidine (Boster Biotech Co. Wuhan, China).

ZO-1, MMP-9 and gp91^phox^ positive staining (brown yellow) was identified under a light microscope. For the image analysis. Ten microscopic fields were randomly selected randomly from each group, imaged at of 400× magnification, and the integral optical density (IOD) for each group was automatically measured using the digital software Image ProPlus 5.0 (Media Cybernetics, USA).

### Real-Time quantitative PCR

Total RNA was obtained from the ipsilateral cerebral cortexes that had been treated with PBS using Trizol reagent (Invitrogen, USA) according to the manufacturer’s instructions. Purity was confirmed by spectrophotometry. The mRNAs were quantified on a Bio-Rad C × 96 Detection System (Bio-Rad, USA) using the Green PCR kit (Fermentas, USA) and gene-specific primers. cDNAs were used as a template for quantitative real-time RT-PCR. β-actin was used as an internal control to normalize the of gene expression levels. The specific primers for ZO-1 MMP-9, gp91^phox^ and β-actin are listed in Table 2. Thermal cycling was initiated with a 2 min incubation at 50 °C, followed by a 10 min denaturation step at 95 °C and 40 cycles at 95 °C for 10 s and 59 °C for 50 s. The relative quantities of the candidate genes and b-actin mRNA were calculated using the comparative threshold cycle (Ct) method.

### Western Blots

For the Western blotting analysis, RIPA buffer (Applygen Technologies Inc., Beijing) and cell lysis buffer for Western and IP (Beyotime Institute of Biotechnology, Jiangsu) were added to the homogenized ipsilateral cortex and collected cultured cortical astrocytes, respectively. Then, PMSF and protease inhibitors were added. The lysate was separated by centrifugation at 12,000 × g at 4 °C for 15 min, and the supernatant was collected. Protein concentrations were determined using a BCA assay kit. Cytoplasmic proteins were diluted in loading buffer, subjected to sodium dodecyl sulfate polyacrylamide gel electrophoresis (SDS-PAGE), and transferred to PVDF membranes. The membrane was blocked with a freshly prepared 5% milk-TBST solution for one hour at room temperature and then incubated with the primary antibodies (the antibodies are listed in Table 1) overnight at 4 °C. After washes in TBS-T (3 times with 15 min for each), the membrane was incubated with the appropriate HRP-conjugated secondary antibody (diluted 1:3,000 in secondary antibody dilution buffer) for one hour at 37 °C. After washes with TBS-T (3 times with 15 min for each), the protein bands were detected with chemiluminescence and exposed to X-ray film. The films were then scanned and the band density was quantified using UN-Scan-It 6.1 software (Silk Scientific, Inc., Orem, UT). The β-actin antibody was used as an internal standard.

### ROS level measurements

For the *in vivo* experiments, the stored cortexes were weighed, dissected and homogenized in 9 volumes (1:9, *w/v*) of ice-cold normal saline in a homogenizer (TissueLyser LT, German). After centrifugation (3000 rpm at 4 °C for 15 min), the supernatants were used to measure the levels of ROS production. For the *in vitro* study, the rat astrocytes were collected to examine ROS production in response to different 24 h treatments.

ROS levels were measured with a dichlorodihydrofluorescein diacetate (DCFH-DA) assay (Beyotime Institute of Biotechnology, Nantong, China), according to the manufacturer’s instructions. Briefly, the cells and tissues were resuspended with PBS and stained with 10 μM DCFH-DA for 20 min at 37 °C in the dark. After the samples were washed 3 times with PBS, lysis buffer was added to the cells and tissues. Relative fluorescence intensity was recorded at the indicated times using a fluorometric imaging plate reader, with an excitation wavelength of 488 nm, and an emission wavelength of 525 nm.

### Statistical analysis

All data are expressed as mean ± standard deviation (SD). The results were analyzed using an unpaired t-test to determine the statistical significance of the treatment sets. Multiple comparisons were analyzed using one-way analysis of variance (ANOVA) followed by the Tukey post-hoc test. The difference was considered significant when p < 0.05.

## Additional Information

**How to cite this article**: Wang, Y. *et al*. Rhein and rhubarb similarly protect the blood-brain barrier after experimental traumatic brain injury via gp91^phox^ subunit of NADPH oxidase/ROS/ERK/MMP-9 pathway. *Sci. Rep.* **6**, 37098; doi: 10.1038/srep37098 (2016).

**Publisher's note:** Springer Nature remains neutral with regard to jurisdictional claims in published maps and institutional affiliations.

## Figures and Tables

**Table 1:** Primary antibodies used in western blot and immunohistochemistry.

Primary antibody	Commercial source	Catalog number	Species	Working concentration
gp91^phox^	Abcam	ab131083	Rabbit	1:1000
p-ERK1/2	CST	4370 S	Rabbit	1:2000
ERK1/2	CST	4695	Rabbit	1:1000
MMP9	Abcam	ab38898	Rabbit	1:1000
ZO-1	Abcam	ab59720	Rabbit	1:50
GFAP	CST	3670 S	Mouse	1:1000

**Table 2:** Summary of the RT-PCR primers sequences.

Gene	Primers	Sequences	Product length
ZO-1	Forward	5′-CGTTTATCGCCGCATTG-3′	201 bp
	Reverse	5′-CCTCGCTCTACCTCCTTGTG-3′	
MMP-9	Forward	5′-GCAAACCCTGCGTATTTCCAT-3′	76 bp
	Reverse	5′-CCATCCGAGCGACCTTTAGTG-3′	
gp91^phox^	Forward	5′-GGAAACCCTCCTATGACTTGG-3′	202 bp
	Reverse	5′-CGGGACGCTTGACGAAA-3′	
β-actin	Forward	5′-CATCCTGCGTCTGGACCTGG-3′	107 bp
	Reverse	5′-TAATGTCACGCACGATTTCC-3′	
